# Reflecting on the Synergy Between Computer-Mediated Intercultural Communication and the Coronavirus Memory Archive: An Intrinsic Case Study

**DOI:** 10.1177/21582440231181298

**Published:** 2023-06-15

**Authors:** Angeliki Ypsilanti, Ioannis Karras

**Affiliations:** 1 Hellenic Open University, Patras, Greece; 2 Ionian University, Corfu, Greece

**Keywords:** coronavirus, memory archive, EFL, computer-mediated intercultural communication, synergy

## Abstract

The coursebook-centered approach adopted by the Hellenic Open University’s Master’s program in TEIL/TEFL has lost momentum during the coronavirus lockdown periods. This study addresses synergies between institutional policy and involved participants through the experience of an EFL teacher with a Computer-Mediated Intercultural Communication-based assignment about the online archivability of teaching and learning reflections. Relying on her study journal, the EFL teacher proposed a reflection-based inquiry into the compilation of a student-generated coronavirus memory archive on the e-me digital platform. Three journaling spaces emerged in the context of teacher development: the physical, the digital and the academic archive. The results indicated the transformative effects that archival procedures have on the dominant training practices of a graduate-level TEFL program. This study holds implications for flipping a switch on the educator-educatee relationship, while examining how reflective teaching pedagogy assimilates the learners’ creative response to remoteness.

The Hellenic Open University (HOU), the only Higher Education Institution (HEI) in Greece that provides open and distance education on all levels of tertiary education, views self-regulation as that “self-directive process by which learners transform their mental abilities into academic skills” (Zimmerman, 2002, p. 65). Although in self-regulated learning (SRL) the HOU’s printed educational material and the module tutor assume a more facilitating role, linearity in course planning and delivery (course content—teaching strategies—student skills) remains teacher-focused. It is the serviceability of this linearity to the pattern relationship (Master’s module tutor—teacher-educatee—school student) in the HOU’s teacher development programs that has been disrupted by the coronavirus pandemic outbreak. As an alternative to the above pattern, a mediative potential has been set up to remedy the consequences of shifting to online teaching, which has become known as emergency remote teaching (ERT).

The contextual background to this case study is the HOU’s Master’s in TEIL/TEFL which is an advanced teacher development program addressed to EFL teachers of the public and private sector. The focus of interest is the Intercultural Approaches to the Teaching of English module which is one of the elective modules offered by the Master’s program in TEIL/TEFL. As the module’s title implies, its relevance is closer to the Teaching of English for Intercultural Communication (TEIC) and/or for Multicultural Awareness (TEMA). Potential participants are invited to “become explorers of the ‘cultural’ and ‘intercultural’ territory and to consider the many different ways in which this territory might aid them as they critically reflect on their professional identity, context, and practices” (Fay, 2008, p. 21). The module is supported by two textbooks dating back to 2008 and revised in 2018 that serve as study guides (Fay, 2008; Fay et al., 2008). The textbooks’ aims, content, and learning outcomes are complemented by a culminating assignment. The rubrics of the assignments are part of the textbook content and cannot be modified or replaced by the module tutor.

During the first school closure which started on March 23, 2020 in Greece, the EFL teachers (to be referred to as teacher-educatees) of the HOU’s Master’s program in TEIL/TEFL had to complete their final assignment for the above-mentioned module. The assignment centered on the development of an effective Computer-Mediated Intercultural Communication (CMIC) project that would cater for their students’ Intercultural Communication Competence (ICC) skills development. The rubric of the assignment was preset by the provided textbook (Fay et al., 2008), which meant that the development criteria would have to conform to the prescribed textbook material. As for the assessment criteria, standard though they were, they supported subjective interpretation. The fact that an unprecedented crisis discontinued the regular pattern by introducing new nonlinear instabilities entailed adopting more flexible standards in terms of work practices.

With everyone’s sense of normalcy disrupted, existing teaching and learning modes went into a holding pattern making experimentation with new schemes of work the overriding concern of the Greek Ministry of Education. The Massive Open Online Course (MOOC) initiative undertaken jointly by the Institute of Educational Policy (IEP) and the Hellenic Open University to provide synchronous and asynchronous education training to teachers of all education levels was carried out by the HOU’s independent unit, the Educational Content, Methodology and Technology Laboratory (e-CoMeT Lab), via the Learn digital platform. What is at issue here is that despite the emphasis on the methodology of online education using the officially approved digital platforms (e.g., WebEx, e-class, e-me), participants were invited, but not provided with a designated space, to either record or share among themselves critical reflections on the study material. In contrast to other emergency coping MOOCs running during the same period such as the “Resilient Teaching Through Times of Crisis and Change by Michigan University,” which made provisions for personal journaling space via the Gamut workbook tool and deliberative forum space via the platform-based discussion tool, teachers in Greek schools were never given the official opportunity to either write down their reflections or interact with each other in shaping common professional development practices. To this end, the present study aimed to explore possible reflection spaces within the official digital working environments. The e-me platform, the first organized attempt at a Personal Learning Environment (PLE) for Greek school education, provided fertile ground for the development of self-regulating synergy among the teacher educator, the teacher-educatee and the school students. The main aim of the study was to use a reflection inquiry-based approach to empower all parties involved to contribute to the implementation of a CMIC-based coronavirus memory archive project that would transform online homework space into a collective workspace.

While the development of a CMIC project did not pose any real challenges as to its integration with the new digital reality, it brought about the ethical dilemma as to its instant execution given the school students’ poor online engagement. The coronavirus-related disruptions epitomized the dilemma over how teachers modulate the way they work in response to their students’ needs, while asserting the value of their effective teaching strategies. For the purposes of the course assignment, one of the teacher-educatees of the graduate program decided to slow down the flow of her online teaching sessions in order not only to refresh her students’ interest in the English lesson, but also to give them space to pause and think about the effects of the new corona reality on self-discipline maintenance. Thus, her response to the CMIC project rationale departed from purposeful tweaks to the assignment’s rubric and culminated in the composition of a Master’s dissertation on the transformative dynamics of the writing skill as a source of reflection and motivation within the setting of a PLE digital platform with social networking ramifications.

The case study was based on 17 high school students’ personal experience essays, a CMIC-based course assignment, a Master’s dissertation, and two feedback evaluation forms to answer the following research question:

How can graduate-level TEFL programs benefit from the self-regulating synergy between the teacher educator, the teacher-educatee, and the teaching sessions during emergency remote instruction due to Covid-19?

## Literature Review

### Defining the Intrinsic Nature of Transferring Online

A large part of this research is aimed at revealing the particularities of “a case within the case” since according to Stake (2003) once a specific case has been preselected for its intrinsic appeal to the teacher as researcher the amount of selectivity required in choosing between “propositional” and “experiential learning” is reinforced by the observational variables of the learning behavior. It is this implied kind of experiential unpredictability that reflects the intrinsically random nature of a case whose added complications are encountered “by chance” (Isaacson, 2016).

Zainal’s (2007) perspective on “single-case design” regarding the preclusion of replication due to an event’s limitation to a single occurrence provided the starting point for the field observation that the serendipitous emergence of the coronavirus-related remoteness variable was singularly disruptive of the transferability from onsite to online learning. In other words, the completion of a post-graduate course assignment under remote learning circumstances became contingent upon the involved parties’ adaptive responsiveness to experimental online settings rendering it a case worth investigating because of its apportioned effects to the corresponding levels of intrinsic interest.

More purposely, “the more the researcher has intrinsic interest in the case, the more the focus of study will be on the case’s uniqueness, particular context, issues, and story” (Stake, 2003, p. 155). The researcher’s own internal motives are thus as important as the subject of the study’s “uniqueness” since perceptual observation and interpretation are correspondingly instrumental in the monitoring and documentation of a case whose developmental transition from ordinary to unique requires as much cognitive as affective skills and practices.

### The Destabilizing Role of Remoteness

There is a lot more to experiencing remoteness than residing in confined regions or having a bad internet connection. Anxiety about having lost one’s access to the physical space of education constitutes a destabilizing force with respect to student online participation. On the issue of space suitability, Eder (2020, p. 169) clarifies that “remote education [ . . . ] necessitates an environment and atmosphere that is conducive to effective teaching and learning.” What happens though when students’ particular living conditions thwart adaptation to new teaching delivery methods? And more importantly, is the measurement of student performance during synchronous teaching sessions an attainable goal for everyone? To preempt the effects of unequal student involvement, Eder (2020, p. 169) proposes that the United Nations’ adopted principle of “doing no harm” be applied to educational policy advocating that “in the case of remote instruction, if only some group of learners will benefit while the rest does not, then it should not be rolled out.” Within the context of the present study, the “doing no harm” principle fits perfectly because the asynchronous environment of the e-me digital platform is proposed as a non-threatening alternative to the emergency WebEx video conferencing service in that it does not exacerbate the inequitable provision of public education. Additionally, the completion of a post-graduate assignment relies on bottom-up goal-oriented behavior as a cue to student mediation services to effective teaching.

### The Affective Component of Computer-Mediated Intercultural Communication (CMIC)

In the same way that the influence of the HOU’s assigned textbook material has been rendered inconsequential because of its lack of synchronization with the new corona reality, the sustainable correspondences between the assigned EFL coursebooks, and the new virtual reality for education will hold only up to a point; that of being intrinsically characterized as short-term retrofitting options. Since the modalities of communication as well as the notion of scopes in web-based instruction are aligned with the students’ life circumstances, a “doing no harm” retrofitting option would be that of selecting the Asynchronous Computer-Mediated Communication (ACMC) version to establish moderate-risk alignments at the level of communication.

An ampliative argument for the benefits of CM(I)C as compliant with ERT comes from Nguyen (2008, p. 28) who explains that “CMC, due to the characteristic of space and time independence, is widely known for affording both intercultural and intracultural exchanges.” Capitalizing on the above assertion, the question is to what extent a compensatory CMIC-based project might overcome the challenges of space and time in a way that the emerging psychological mediators of disruption can be well accounted for. Part of the answer could be traceable among Hauck’s (2007) own challenges to internet-mediated, intercultural foreign language projects, markedly in the one associated with the exploration of the affective stimuli to student response. At the same time, all of this closely parallels Karras’ (2020) perspective of empathy as the adjacency of two components—the cognitive and the affective—subscribing to the ICC paradigm. Awareness of such conceptual parallels triggering fortuitous matches across different contexts can then help to advance research in the affective components by means of the insights gained from each of them.

Making space and allowing time for EFL students to regulate their adjustment to the volatility of the situation entailed providing them with affective motives for keeping pace with online learning. Part of the directional hypothesis was that prompting empathetic attunement to the simultaneousness of a global crisis would help students to engage in interactive discourse practices as a way of legitimizing their coronavirus-derived status of remoteness. The authorization to communicate the emotional awareness of the physical distance between sympathizers is this paper’s answer to the parametric instabilities of the coronavirus education period.

### Recording the Conceptual Development of Reflective Journaling

St. Amant (2002) explored the “points of contention” between CMC and intercultural communication putting forward his concern about the different scopes in negotiating online identities. The bone of contention was CMC-oriented fluidity of agents’ identity which was inconsistent with the stability required for the purposes of effective intercultural communication. From then until 2018, the “points of contention” have been resemanticized as “points of friction” which affect the trusting relationship between the agents and the targeted audience (Rice & St. Amant, 2018). In both cases, negotiation of intended meaning is mediated through written content featuring in global cyberspace while, in general terms, the “contacting, conveying and connecting approach” is touted as the panacea to “the complexities of audience and purpose” (Rice & St. Amant, 2018, p. 9).

With the advent of the coronavirus pandemic in 2019, the above points regarding identity communication were highly skewed since the CMC focus shifted to the development of joint project implementation through “digital notebook” technologies with open access (Walwema, 2018). While Walwema (2018) argued in favor of collaborative writing practices in digital spaces to enhance global dialogue among peers working toward the requirements of a composing task, reflective feedback received less prominence with respect to output practices, thus thwarting efforts to reach a middle ground on the issue of authoritative (teacher-oriented) versus non-authoritative (learner-oriented) discourses (Vujaklija, 2021, p. 653). Archiving learner-generated inputs and teacher-generated outputs constitutes a CMIC proposal to construct and reconstruct reflective practices for future reference.

Because digital notebook resources are also operable in the PLE environment of officially approved digital platforms, teachers, and learners can experiment with the transformative possibilities of the storage space facilities to engage in reflective journaling practices. Vujaklija (2021) pinpoints that participating in reflective writing is a pause-to-think-and-feel strategy that paves the way for the “ideological becoming” of teachers and students seeking to conceptually legitimize a new course of classroom practice through the development of non-authoritative discourses of self-representation. Within the context of the present study, EFL teachers and students contextualized their thoughts and emotions via empathetic responses to the reflection prompts of their corresponding statuses and spaces.

### Toward an Understanding of a Coronavirus-Reflecting Pedagogy

Adaptation to the new coronavirus educational reality poses a real challenge since aims and objectives of distance education as conventionally perceived seem outdated. Before adaptation becomes a realizable goal, a renewed synthesis of several component reflection processes must come into effect. For a better understanding of the role of reflective practice in education, it suffices to conceptually break free from Gibbs’ (1988) performance-based cyclical pattern of reflection and draw on the behavior of reflection in Physics for the sake of an alternating pattern. If we stop for a moment to think how the continuum spectrum of coronavirus as a source of diffused light reflected upon people’s lives, the question that arises is how its consequences have been absorbed and reflected in students’ thoughts and feelings. In the present study, students composed pieces of self- reflective writing in the same way that physical objects’ surfaces re-emit a linear spectrum at various frequencies. The teacher-educatee in another dual role, as teacher-researcher this time, used her spectroscope to observe and analyze the linear spectrum only to find out that the student-reflected memory archive re-emitted at relative frequencies which became her provisional data to use through an assignment-prompted observational procedure. The bottom line is that the sending and receiving need of inserting and extracting meaning in and from spectroscopic observations is not static but rather ongoing. Therefore, sequentially, archiving coronavirus-prompted reflections becomes, first, a student-generated and, second, a teacher-oriented task whereby “the archive as prosthesis analogy sees memory being prompted by the archive but also conceives the archive as something created by memory” (Brown, 2013, p. 89).

Hence, the questions inherent in the main research question: Are remote students “safe” in the case of teacher-prompted archival of reflection on experience? If so, how does this archived mode of reflective practice that forms an imperfect measure of the actual incidence of the coronavirus pandemic’s impact on education delivery methods facilitate the online synergy between the teacher educator, the teacher-educatee and the school students?

## Study

What follows is matching of archivable context and corresponding participants within an in-action-on-action timeframe provided by the reflective processing of the collected data. The discussion of the findings, based on the emergent themes, brings in the study’s implications, limitations, and directions for further research presented in a comprehensive conclusion.

### Method

Given the above concerns, this intrinsic case study sought to disengage in-action reflective practitioners from clutching onto anticipated recurrences of stimulus properties by drawing on either qualitative or quantitative measures. Conversely, freed from the clutches of Gibbs’ (1988) iterative cyclicity and Hall’s (1997)“deliberate” systematicity—concepts clearly geared toward the replicability of established reflective frameworks—this study’s record-kept reflective practices acquired intercultural, ideological, and practical dimensions in response to and within the re-established parameters of coronavirus teaching and learning. This study verges on Schön’s (1987) dichotomized understanding of reflection-in-action-reflection-on-action as a complete reflective pedagogy model. The laid-out reflections, either assigned or developed as affective tasks, are a part of the while-learning and -teaching process.

### Participants

This case study focused on the pool of 17 high school students who got involved in a CMIC-based project administered by the EFL teacher of a Lyceum in Greece for the purposes of the completion of a postgraduate course assignment. The design of the assigned project, whose performance standards had to be regulated during the first school closure in March 2020, became the teacher-educatee’s reason for producing associative links between the upper tier (HOU’s module tutor) and the lower tiers (teacher and students). To that end, all involved parties became mutually involved in challenging rubric specifications by removing what they regarded as impediments to equitable progress. Appendix A provides short profiles for each of the participants.

### Context

Although participant forechoice was a given owing to the three-tier system of interconnections involved in the advanced teacher development program in question, it matured into an alliance of convenience when it all came down to strategizing between top-level forward mapping (conventional regulation of assignment design) and ground-level backward mapping (reflective prevention of the achievement gap). To that end, drawing level became everyone’s practical and ethical preoccupation via prompted reflection in their assigned spaces. Google Docs, succeeded by e-me platform, pen-and-paper study journal and feedback forms became the primary sources for the identification of three different types of archival spaces: the digital archive, the physical archive, and the academic archive.

### Data Sources

The data came from seventeen students’ personal reflection essays archived on the e-me digital platform that received teacher and peer feedback through blogging during the second school closure in Greece (from November 9th to February 15th, 2020), the teacher-educatee’s fourth assignment for the Intercultural Approaches to the Teaching of English module and Master’s dissertation for the Master’s program in TEFL/TEIL, and the module tutor’s/dissertation supervisor’s evaluation of both.

### Data Analysis

The data analysis process was in-action reflection throughout the study. The data were coded into emergent themes through on-action reflection during which the ethical ramifications of ERT and SRL were considered in relation to the care-centered approach. The descriptors of the seven themes that have emerged from the data are found in Table 1. The themes defined in Table 1 are as follows: Self-care Writing Patterns; Concluding Contemplating Remarks; Digital Collaboration Writing Skills; Social Networking Features of PLE; Archived Coronavirus Memories; Shifting Marking Criteria; Learning Circumstances in Times of Crisis.

**Table 1.** Emergent Themes From the Case Study Data.

*School students’ reflective essays*
1. Self-care Writing Patterns—students’ references to changes in daily routines and habits due to remoteness 2. Concluding Contemplating Remarks—students’ direct address to the local and global reader
*Teacher-educatee’s course assignment and Master’s dissertation*
3. Digital Collaboration Writing Skills—teacher’s discussion about the reflection spaces as fulfilling the components of ICC 4. Social Networking Features of PLE—teacher’s ideas about their contribution to transformative learning 5. Professional Practice—teacher’s discussion of the archived coronavirus memories and their implications of use in the online teaching setting
*Master’s module tutor’s/dissertation supervisor’s evaluation*
6. Shifting Marking Criteria—tutor’s discussion of shifting specifications of rubrics in exceptional cases 7. Learning Circumstances in Times of Crisis—recognition of the academic impact of Covid-19 on teacher research

## Findings

### The Physical Archive

The first (ground) level of the study was textbook-prompted reflection-in-action which involved the EFL teacher’s recorded response in her pen-and-paper study journal to the “Self-Reflection Prompt 10.15a: During and after the meeting, think about how the synchronous space affects the way you communicate” (Fay et al., 2008, p. 200).

Prior to the completion of a course assignment, “thinking through a CMIC project” was the focus of the relevant online course session concomitantly with self-reflecting on the pros and cons of synchronous communication depending on the provided space (Fay et al., 2008, p. 217).

#### Reflection one (during the meeting)

Angela (the teacher-educatee) wrote the following journal entry during the online meeting with the module tutor:

I need to go through the textbook information on CMIC project rationale and work through my own by drawing upon the model provided. An important point to keep in mind is that computer mediation affects communication in unexpected ways that may not be under one’s supervisory control. On the one hand, silence or non-communication seems to be an unsurpassable hindrance to synchronous communication. On the other hand, among Hauck’s challenges to CMIC project rationale, the reported affective variables hold a central position since without “motivation” or “willingness to communicate,” implementation seems condemned to failure from the outset.

#### Reflection two (after the meeting)

After the online meeting with the module tutor, Angela proceeded to the following journal entry:

Students cannot keep up with the pace of the lesson if there are no open channels of communication with their teachers during school closure. With email being the only viable solution for the time being, development of ICC seems almost out of the question since not everyone looks disposed to become tuned to the latest technological advancements of CMC. What is worse, lack of motivation has deprived almost everybody of the stamina to progress beyond survival mode. A good idea would be to make students aware of coronavirus experiences that are universal in order to activate empathy and sympathy as ways of regulating unprocessed emotional stress. To allow space and time for processing and externalizing accompanying problems, maybe pausing for a short while to rewind would be preferable to continuing with the standard syllabus.

#### Digital collaboration writing skills

“[ . . . ] pausing for a short while to rewind would be preferable to continuing with the standard syllabus.” The teacher-educatee’s journal entries, far from buttressing the silence argument, they merely added to the inquisitiveness into flexible spaces of CMC. In this context, pausing has meant disengaging from the linear logic of the coursebook content while attending to the availability of compensating options. As a first step, the EFL teacher flipped textbook-dependence (hence the disengagement from authoritative sources and discourses) and exerted bottom-up processes regulable in an extra-curricular context. Hence, digital collaboration writing on Google Docs became the learner-directed pause-coping option that served the purposes of a digital notebook that “[ . . . ] opened up [ . . . ] global communicative possibilities for students” (Walwema, 2018, p. 16).

At the same time with having recourse to retrospective modification of the assignment’s rubric, the teacher-educatee accommodated Hauck’s four challenges to a real-world design of a CMIC project and came up with her own applicability conditions establishing a one-to-one relation between an ICC component and a universally functional determinant in the CMIC equation. The fused result (adapted from Karras, 2020, p. 104) can be found in Appendix B. Placing special emphasis on the emotional limbo state in which the students inadvertently found themselves, the teacher-educatee experimented with Google Docs for its document authoring facilities to give voice to students’ personal views of the coronavirus experience.

#### Social networking features of PLE

“[ . . . ] lack of motivation has deprived almost every one of the stamina to progress beyond survival mode.” Experimentation with Google Docs was followed by training on the social networking features of a PLE. With the educational process itself delegating accountability to promote the salience of lower-tier, non-authority discourses, the Google Docs free web application was replaced by e-me platform, the first organized attempt at a PLE for Greek school education. On e-me, students had to submit a term assignment on their coronavirus experiences to be used as archivable material for the assemblage of a collective memory archive. This constituted a watershed moment in students’ motivation to press on because they were granted both private and public space to self-manage and -monitor while embracing the opportunity for transformative, impactful learning.

The end written result of the students’ built-up reflection through the opened channels of communication provided the teacher-educatee with the resources and the focus to process and tailor a CMIC-based project that entailed its conformance to the pandemic-induced constraints. The interim reflection spaces were accordingly utilized for transformative teaching and learning purposes. The social networking features of e-me, that is, the blogging facilities that supported computer-mediated intercultural communication, contributed to the maturation of the teacher-educatee’s end-of-module assignment into a Master’s dissertation.

#### Professional practice

Teacher-guided classroom communication seldom produces unanticipated results because losing face is a calculable deterrent. However, “[ . . . ] computer mediation affects communication in unexpected ways that may or may not be under one’s supervisory control.” On the research front, the derived outcome, based on the students’ reflections on remoteness repercussions on their learning progress, asserted the teacher’s hypothesis that professional practice can benefit from the self-regulating synergy between the teacher educator, the teacher-educatee, and the teaching sessions during emergency remote instruction due to Covid-19. It could be argued that her assignment reflected her recorded conclusions in her study journal while her Master’s dissertation constituted on-action-reflection on the improvisational planning of the CMIC-based project.

### The Digital Archive

The second (ground) level of the study was the assemblage of the coronavirus memory archive on the storage space of e-me, the Hellenic digital educational platform’s “hives” which “accommodate smaller, self-contained social, regulated learn-places” (Megalou & Kaklamanis, 2018, p. 150). Part of the hives’ substructure is “e-me assignments” where students can make individual submissions for teacher feedback and “e-me blogs” where students and teachers can share content and interconnect.

#### Self-care writing patterns

Remarkably, even though coronavirus remoteness affected each student differently, all of them used its repercussions for their life to good effect. Below are some of the students’ statements.

About his day-to-day life, Stratos observed:

At this point, my daily routine changed radically. I couldn’t go to school anymore and therefore lessons were delivered online. [ . . . ] My only means of communication was from my computer. It seemed like time had stopped. I was under a lot of pressure, anxious and stressed for my parents and grandparents. Nevertheless, I always hoped for the best and tried to take advantage of my free time to study more and come closer to my parents.

On the issue of self-care, Kostas went so far as to say:

At the beginning of the quarantine, I was miserable, bored out of my mind, and sad. I still made the time to deal with the load of work that was thrown my way, while also finding the time to enjoy myself by doing what I wanted. I started working out, listening to music, and watching new movies.

Despoina used nature- and family-bonding as the best solution to social-distancing:

The fact that I live in a town close to nature and the opportunity to walk by the sea everyday was the best escape for me. [ . . . ] Moreover, it was spring, and everyone was walking by the sea [ . . . ]. I can’t say that it had a bad effect on our family since we had the opportunity to spend more time together.

#### Concluding contemplating remarks

Having gone through the whole range of emotions after the initial shock of being led into emergency mode, students came to the realization that they were responsible for taking care of themselves so that they did not squander the provided opportunities. This turn to self-care catalyzed the need for making a statement about the impact of coronavirus on their life priorities.

Taxiarchis saw coronavirus as the vehicle for bringing about creativity and optimism in his life: “I always try to make my day more creative and to banish negative thoughts and feelings.”

Dimitra gave her own take-away for co-suffering readers: “I believe that all of us have learned a lesson: not to take anything for granted and to appreciate everything in our lives.”

Apostolis managed to establish a new set of priorities: “I have realized some changes in my thinking on various issues. Health and freedom are important for human existence.”

The compilation of the school students’ reflective essays was initially piloted through email and on Google Docs to establish their intending-foreseeing quality in view of their archival usability on e-me. The students’ self-referential writing disposition meshed constructively with the dynamic context of the memory archive while the negative pole of remoteness was compensated for by the publishable potentiality of the submitted content. Email, digital writing on Google Docs, and final submitted assignments on e-me gave students the opportunity to finesse their reflective writing skills by producing a memory archive of the while-Covid-19 era. The fact that each writing piece started with a self-care pattern and ended on a contemplative note lent the students’ work a global level of peaking synchronization.

### The Academic Archive

On the tutor’s/supervisor’s receiving end, the feedback and evaluation form became a specific space for conveying self-reflection which rendered what may have started as suggestive and speculative ethically acceptable and academically stable. Building his feedback of Angela’s module assignment and Master’s dissertation, John (the tutor/supervisor) integrated academic, ethical, and practical considerations in his role as legitimizing agent. Maximizing on the long-run practical implications of his feedback, the teacher-educatee recognized a key attribute being that of care-oriented flexibility. What will follow are the module tutor’s feedback comments on the teacher-educatee’s end-of-module assignment and final year dissertation construed in either of two ways: as authoritative activation of an affective domain in assignment evaluation (through shifting marking criteria) or as qualitative foresight into upcoming educational change (through acknowledging learning circumstances in times of crisis).

#### Feedback and evaluation form (Assignment 4)

John uploaded the following feedback and evaluation form on the HOU’s study portal:

Dear Angela,

I’m glad to see that you have maintained a high level of quality in your work. You have repeated some issues that I pointed out last time. I understand that your level of thinking/analysis/reasoning is quite high, but to be fair to everyone, you need to conform to the assignment specifications. I’m still assigning a good grade because of your hard work. Having said that, your approach to the current pandemic, was interesting. I would encourage you to look into doing a dissertation in this area. Feel free to call me so we can discuss this further if you are interested.

Keep up the good work!

#### Feedback and evaluation form (Dissertation)

John completed his feedback by concluding: “This was an interesting dissertation to read, especially with the current situation re COVID. The findings generate interesting implications for practitioners and students alike.”

#### Shifting marking criteria

In accordance with the HOU’s analytic marking guide, an objective sub criterion within the “Content” criteria category is “application of principles to practice.” The supervisor’s alternative activation of a subjective perspective constitutes a specific endeavor to reveal both the objective and subjective implications in a manner that is ethically just for educators and educatees alike. The assignment of a good grade despite non-conformance to rubric specifications constitutes a substantial shift in marking methods.

“I understand that your level of thinking/analysis/reasoning is quite high, but [ . . . ] you need to conform to the assignment specifications. I’m still assigning a good grade because of your hard work.” Noddings (1995) describes the four elements of his proposed care-centered model of education, one of them being that of “confirmation.” Transferring the care ethic to an online context, Robinson et al. (2020) elaborate Noddings’ perspective on confirmation by drawing on Buber’s (1970) earlier work where *confirmation* is viewed as the observer’s positive stance reflecting on what or who is observed. The fact that the assignment’s outcome was highly graded despite its non-conformance to rubrics’ specifications was a gauge of its resonance with the tutor/supervisor/evaluator. In this framework, the established resonances—positive feedback loops that bound the developing project’s stages into a coherent whole (Master’s dissertation) provided confirmatory evidence of how three distinct occupational roles (tutor, supervisor, evaluator) integrated affective functions (empathy, flexibility, sensitivity) with cognitive functions (“thinking,” “analysis,” “reasoning”).

#### Learning circumstances in times of crisis

“I would encourage you to look into doing a dissertation in this area.” [ . . . ] This was an interesting dissertation to read, especially with the current situation re COVID. The findings generate interesting implications for practitioners and students alike.” Although in Noddings’ (1995) care-centred model “confirmation” comes after “practice,” in this case, practice followed confirmation. Only after grappling with and accommodating her teaching and learning style to implement the CMIC-based module assignment did the teacher-educatee receive reaffirmation to convert her memory archive-related idea on Google Docs into organized research using an official digital platform to host the student-generated archive. Pre-composition, practicing the act of caring has entailed allowing (teacher and school students) sufficient time to reflect on individual and shared experiences with corona teaching and learning before going public with (uploading) them for others to view and process (Robinson et al., 2020).

## Discussion of Findings

This case study, the centerpiece of which was spaces for reflection, proposed the archival arrangement and description of the students’ coronavirus memories as a showcase example of distance education innovation. More pointedly, innovation had to do with building on students’ elevated sense of agency to document the synergistic relationship between the university educator, the teacher educatee and the school students with the aid of new tools and ideas. Intrinsically linked to the compilation sequence was Covid-19 which was given significant thrust through the development of a self-regulating emergency plan.

As a result, the shift of focus on learning as a one-way process in which students are conventionally passive recipients of knowledge to learning as a reciprocal process through which students actively reflect on the way knowledge affects them was practically a co-authorship type of proposal in a Students as Partners (SaP) type of context. On the issue of student-faculty partnership, Mercer-Mapstone et al. (2017) argue that assuming shared responsibility in order to prevent inequality in knowledge distribution should reflect not only in co-inquiring about the whys and wherefores but also in co-documenting results. In this case study, partnership has meant passing on the baton in a reflection process that took effect the moment Covid-19 was interpreted as a useful occasion for rehearsing what Cook-Sather and Felten (2017) call the “ethic of reciprocity” in pedagogical relationships. The resultant archive became the created “space” and “opportunity” for reciprocal commitment to a form of targeted intervention into its “exploration” and “growth” requirements (Cook-Sather & Felten, 2017, p. 5).

The use of the memory archive as a safety valve to release emotional tensions and combat inequalities in online course delivery methods became operable within a PLE context, underwent structural adjustments in its transition to a reliable vessel that steers counter-hegemonic ideas into ascendancy and imparted a “care-for-others-and-balance-service-and-needs” philosophy either upwards or downwards on the hierarchy scale (Mele, 2003). Mere consistency maintenance between the affective and the cognitive aspects of CMIC has meant performance accountability ranked second to the need for enhanced resilience to change. By and large, this intrinsic case study indicated that reflection-in-action weighed heavily on all parties involved in the educational process making for alterable strategies along the way. With regard to harm reduction, García and Weiss (2020, p. 26) report on the necessity of embracing such an adaptation strategy that involves the decision of substituting “traditional standardized tests” with alternative types such as “project-based assessments” and “capstone projects.”Dickinson (2020) explains how project-based learning ideas can culminate in term assignments where K-12 students can show off accumulated experience and skills. The digital archive could be regarded as a capstone project because it was such a case of a term assignment that encapsulated experience, ability, and creativity.

Still, when it comes to deciding upon adaptation strategies, how do we account for equitable access? The present study highlights the need for synergizing and strategizing at the same time and at the same level depending on the circumstances. This case-by-case approach to selecting synergies and strategies to maximize effect, albeit without formally assessing performance, applies to the occupiable spaces for reflection in which may or may not lie ample room for engaging in constructive dialogue via interactive feedback processes. It is worth noting that although the synergistic benefits of the physical, the digital and the academic archive have been construed as the outcome of random contact sequence, reflection-on-action relied on a post-access, recognition process to infer user-context features from aspired self-representations. Hence, causal inference from the eventual formation of a meaningfully and structurally coherent archival discourse was implemented in the aftermath of the CMIC-based project.

Downey and Clandinin (2010, p. 390) explain that “reflective inquiry focuses in on a particularly problematic situation [ . . . ] honing our more generic ability to solve problems.” The fact that this intrinsic case study tried to capture the evolution of “unexpectedness” in a crisis situation such as the Covid-19 pandemic entailed that reflection was construed as an on-the-spot solving option that could have easily been substituted with an alternate one. However, because uncertainty became the main component of the pedagogic relationships in question, the developmental stages of the archive (physical, academic, digital) reflected the conscious dismissal of other potentially “harmful” options.

This study was based on the premise that for an advanced teacher development program to be efficacious, participatory solutions involving student consultation and shared level planning in all directions would be the tactical advantage offered by a distance education model that is self-regulatory by definition. The study showed that rigorous control over course material dependence is less of a concern without practice collaborators during times of crisis. In contrast, ongoing observation for independent variables, whose impact-related behavior may be disrupting as is the case of Covid-19, has manifested the need for the activation of the diffuse mode of thinking as an intuitive defense mechanism against the focused mode of thinking.

### Limitations

Obviously, this study would have benefitted more from comparative discourse analysis of each archive’s reflections as this would have allowed perceptual processing of the successive frameworks within which agencies have operated. Furthermore, the aim of the fulfilment of each space’s educational potential would have been set alongside the co-aim of cross-checking requirement fulfilment. However, the intrinsic nature of this case study has resisted generalized corollaries under the broad heading of reflective inquiry since the scope of inquiry in this paper has been the sustenance of digital platforms as resilient, adaptable, and active agents in fighting off the self-fulfilling prophesies of non-attendance and underachievement. For sustenance reasons under unprecedented circumstances, archiving accountability was set in motion because, ultimately, record-keeping legitimacy became the overriding force that motivated conscious engagement.

### Directions for Further Research

Despite its limitations, this case study raises important points that need to be considered in relation to the establishment of a new coronavirus pedagogy that rejects mainstream behavior as functionally defective in a technology-mediated setting. Not only has the global glossary of pedagogical terms been enriched to signalize the switch from onsite to online learning but learner engagement terms are also being constantly negotiated to anticipate accessibility issues before they arise. With computer mediation, the behavioral result of ICC-targeted projects is facilitated. With combined human mediation, the facilitated behavior can be either goal-oriented or, alternatively, non-goal oriented. Whichever the case, the result of the students’ diffuse thinking has been archived and handed over to its expert beholders for focused interpretation and further diffusion.

## Conclusion

Identifying spaces for reflection has entailed defining the differences in scope between the two MOOCs that the teacher-educatee had attended as part of her professional development practice, the Greek one being a user-friendly guide for the digital platforms provided by the Greek state, whereas the American creating fresh opportunities for self-reflection and self-regulation as prominent co-predictors of resilience in theory and practice.

The remaining question is whose reflection is at stake, the module tutor’s, the teacher-educatee’s, or the school student’s. The answer is simple and complex at the same time since it is only fair to say that all parties have engaged in reflective practices and have arrived at negotiable conclusions. More specifically, the teacher-educatee has synthesized the conclusions about the progress of her assignment on the CMIC-based project and has recorded them in her own study journal. By grounding her line of reasoning on resilience, she has spaced out both her teaching and learning mode to allow change to ingress. By investing in the communicative results of her students’ archived personal experience essays, she has espoused flexibility in her teaching strategies as a showcase of her personal advancement with coping mechanisms regarding outdated assignment rubrics. Her skillful negotiation of the meaning of the assignment rubric by adding to or subtracting from its targeted effect as well as its subsequent upgrade to a globally meaningful outcome with a local point of departure has contributed toward the proposition of an effective reflective pedagogy that operates in reverse. In which case, the inspiration stems from the students and is reflected back on them.

## Appendix A

### Participant Profiles

Angela (whose real name has been changed) is an English language teacher at a Greek Lyceum on an island in the North Aegean. She had over sixteen years’ teaching experience when she was enrolled on the HOU’s Intercultural Approaches to English Language Teaching module. Her final course assignment about the development of a CMIC-based project served as a springboard for her Master’s dissertation *Transforming the EFL classroom into a globalized context: A creative nonfiction narrative inquiry into a Greek virtual learning community during the COVID-19 pandemic*. The emergency remote teaching circumstances during corona times led her to develop her third grade’s students’ term assignments into archivable material to be used for further reflection on coronavirus pedagogy. Her regular contributions to Oxford University’s OCLW’s archival project Life-Writing of Immeasurable Events (LIVE) provided the general framework for a school-based archival project of her own.

John (whose real name has been changed) is the Module Tutor/Theses Supervisor on the Master’s Program Teaching English as a Foreign/International Language at the Hellenic Open University. He had been a postgraduate student himself of the above Master’s program. The title of his Master’s dissertation was *The importance of learning styles and teachers’ awareness of them within an ESP context*. His early resilient teaching mentality can be tracked into one of his Master’s dissertation’s concluding remarks that “teachers are there to help students increase their own learning potential and not prescribe modes of learning suitable to the educator” (Karras, 2002, p. 74). Ten years later, his facilitative stance toward ESP/EAP teaching is evinced in the conclusion of his PhD thesis where he explains that “facilitation” means considering both the affective and cognitive needs of the students (Karras, 2012, p. 300).

Stratos, Kostas, Despoina, Taxiarchis, Apostolis, and Dimitra (whose real names have been changed) were 6 of the 17 students of the third grade of the Greek Lyceum. The coronavirus pandemic caught up with them while they were preparing for the Panhellenic university entrance exams in June 2021. Their responsiveness to the compilation of a coronavirus memory archive through their registration on e-me exclusively for their English class was nearly homogeneous.

## Appendix B

### The ICC Equation

This research was conducted while Angeliki Ypsilanti was at the Hellenic Open University. She is now at Ionian University and may be contacted at angypsilanti@ionio.gr.
